# A novel long non-coding RNA AC073352.1 promotes metastasis and angiogenesis via interacting with YBX1 in breast cancer

**DOI:** 10.1038/s41419-021-03943-x

**Published:** 2021-07-03

**Authors:** Xue Kong, Juan Li, Yanru Li, Weili Duan, Qiuchen Qi, Tiantian Wang, Qifeng Yang, Lutao Du, Haiting Mao, Chuanxin Wang

**Affiliations:** 1 Department of Clinical Laboratory, The Second Hospital, Cheeloo College of Medicine, Shandong University, 250012 Jinan, Shandong China; 2 Department of Breast Surgery, Qilu Hospital, Cheeloo College of Medicine, Shandong University, 250012 Jinan, Shandong China; 3 Pathology Tissue Bank, Qilu Hospital, Cheeloo College of Medicine, Shandong University, 250012 Jinan, Shandong China; 4 Tumor Marker Detection Engineering Technology Research Center of Shandong Province, Jinan, Shandong China; 5 Tumor Marker Detection Engineering Laboratory of Shandong Province, Jinan, Shandong China; 6 The Clinical Research Center of Shandong Province for Clinical Laboratory, Jinan, Shandong China

**Keywords:** Breast cancer, Cell invasion, Long non-coding RNAs

## Abstract

Breast cancer is the major cause of cancer death worldwide in women. Patients with metastasis have poor prognosis and the mechanisms of breast cancer metastasis are not completely understood. Long non-coding RNAs (lncRNAs) have been shown to have crucial roles in breast cancer development and progression. However, the underlying mechanisms by which lncRNA-driven breast cancer metastasis are unknown. The main objective of this paper is to explore a functional lncRNA and its mechanisms in breast cancer. Here we identified a novel lncRNA AC073352.1 that was significantly upregulated in breast cancer tissues and was associated with advanced TNM stages and poor prognosis in breast cancer patients. In addition, AC073352.1 was found to promote the migration and invasion of breast cancer cells in vitro and enhance breast cancer metastasis in vivo. Mechanistically, we elucidated that AC073352.1 interacted with YBX1 and stabilized its protein expression. Knock down of YBX1 reduced breast cancer cell migration and invasion and could partially reverse the stimulative effects of AC073352.1 overexpressed on breast cancer metastasis. Moreover, AC073352.1 might be packaged into exosomes by binding to YBX1 in breast cancer cells resulting in angiogenesis. Collectively, our results demonstrated that AC073352.1 promoted breast cancer metastasis and angiogenesis via binding YBX1, and it could serve as a promising, novel biomarker for prognosis and a therapeutic target in breast cancer.

## Introduction

Breast cancer (BC) is the most common malignancy and the leading cause of cancer-related death in women worldwide [1, 2]. Of late, the age-standardized morbidity and mortality rates in those with BC have been rising in China [3]. Although BC is a molecularly heterogeneous multi-factorial disease [4, 5], studies have indicated that mortality levels are closely related to metastasis, which occurs in >90% of deaths [6]. It is known that tumor metastasis is a complex and multistep process, mainly involving migration, invasion, epithelial–mesenchymal transition, and angiogenesis, as well as other processes [7, 8]. However, the mechanisms of BC metastasis are still not fully understood. Therefore, it is of great importance that the underlying molecular mechanisms of pathogenesis in BC metastasis are studied and that novel therapeutic targets are identified to improve the prognosis of BC.

Long non-coding RNAs (lncRNAs) are a class of non-coding RNAs that are >200 nucleotides in length [9, 10]. New studies have shown that lncRNAs play a critical role in a wide range of cellular processes that could regulate multilevel gene expression, including transcription, posttranscriptional alternative splicing, protein translation and modification, protein transport, localization, and more [11–15]. Abnormal expression of lncRNAs is closely related to the occurrence, development, metastasis, and drug resistance of malignant tumors, including BC [16–18]. For instance, Niu et al. found that a lncRNA, RAB11B-AS1, promoted hypoxia-mediated angiogenesis and BC metastasis driven by hypoxia-inducible factor-2 [19]. Furthermore, Liang et al. demonstrated that a novel lncRNA, BCRT1, promoted BC progression by activating the miR-1303/PTBP3 axis [20]. Moreover, recent reports have revealed that lncRNAs can also be packaged into exosomes to participate in intercellular communication in BC metastasis and progression [21, 22]. Thus, such studies suggested that lncRNA may be critical in the pathogenesis of BC and provide new molecular targets for BC treatment and prognosis.

In this present study, we intended to investigate function and related mechanisms of lncRNAs in BC metastasis. We identified that a novel lncRNA, AC073352.1, was significantly upregulated in BC tissues. High expression levels of AC073352.1 were associated with poor prognosis in patients with BC. Functionally, we found that AC073352.1 promoted BC metastasis and invasion in vitro and in vivo. We further explored the potential molecular mechanism and revealed that AC073352.1 directly bound to the YBX1 to stabilize its protein, which led to the induction of BC metastasis. Additionally, we found that AC073352.1 could be packaged into exosomes and induce angiogenesis. Together, these findings demonstrated that AC073352.1 exerted tumorigenic potential and may be a useful prognosis biomarker and therapeutic target in BC.

## Materials and methods

### BC patient samples, cell lines, and cell culture

All human samples were obtained surgically from BC patients from whom we received informed consent at the Qilu Hospital of Shandong University. The selected patients had not received preoperative chemotherapy or radiotherapy. Fresh breast tumor and adjacent normal tissues were frozen in liquid nitrogen and stored at −80 °C before being used for microarray analysis. Our study was approved by the ethics committee of Qilu Hospital of Shandong University.

Human BC cell lines (MDA-MB-231, MCF-7, BT549, MDA-MB-468, and HCC1937), a normal human mammary epithelial cell line (MCF-10A), HEK293T, and human umbilical vein endothelial cells (HUVECs) were purchased from the Cell Bank, Type Culture Collection Committee, Chinese Academy of Sciences (Shanghai, China). Cell lines were maintained using standard media and conditions. MDA-MB-231, MCF-7, MDA-MB-468, HEK293T, and HUVECs were cultured in Dulbecco’s modified Eagle’s medium (DMEM, Gibco) supplemented with 10% fetal bovine serum (FBS, Gibco). BT549 and HCC1937 were maintained in Roswell Park Memorial Institute (RPMI, Gibco) 1640 medium supplemented with 10% FBS. Finally, MCF-10A was grown in DMEM/F12 (Macgene) medium containing 5% horse serum, 100 ng/ml cholera toxin (Macgene), 20 ng/ml epidermal growth factor, 0.5 µg/ml hydrocortisone (Macgene), and 10 μg/ml insulin (Macgene). All cell lines were grown at 37 °C in a 5% CO_2_ cell culture incubator.

### LncRNA microarray analysis

Total RNA of six paired sets of tissue from BC and adjacent normal tissues were extracted using TRIzol (Life Technologies, USA) and purified using the RNeasy Mini Kit (Qiagen, Germany). RNA quality and quantity were measured by an Agilent Bioanalyzer 2100. The microarray analysis was carried out by Agilent technologies Inc. at Sinotech Genomics Corporation. Briefly, total RNA was checked for RNA integration by the Agilent Bioanalyzer 2100 (Agilent technologies, USA), and RNA samples were synthesized to biotinylated cRNAs for the Sino Human ceRNA array V3.0. Next, the biotinylated cRNAs were hybridized with the slides and the processed slides were scanned with an Agilent Microarray Scanner (Agilent technologies, USA). The acquired array images were analyzed by the Feature Extraction software 10.7 (Agilent technologies, USA). Finally, raw data and heatmaps were produced using the R software package, and the aberrant genes were selected by fold change >2 or <0.5.

### Tissue microarray (TMA) and in situ hybridization (ISH)

The expression level of AC073352.1 in tissues was detected by ISH using a specific digoxigenin-labeled AC073352.1 probe on TMAs, which contained 137 paraffin-embedded BC samples and 67 paired adjacent normal samples. The tissues of BC patients were obtained with detailed clinicopathologic features, including age, gender, tumor–node–metastasis stage, etc. (Qutdo Biotech, Shanghai, China). All of these tissues were sliced at a thickness of 4 μm, and the diameter of each tissue core is 1.5 mm. Further, the quantitative scanning approach was taken to analyze the staining and expression of AC073352.1 using a Nikon microscope. The ISH score was calculated by multiplying the value of intensity of positive staining by the proportion of positively stained cells. An ISH score of <0.5 represented low expression, while ≥0.5 indicated high expression. The sequences for the probing of AC073352.1 are listed in Supplementary Table 1.

### Small interfering RNA (siRNA) transfection

Three individual siRNAs were used to knock down AC073352.1 and YBX1, these were designed and synthesized by Gene Pharma (Shanghai, China), and the two with the best efficiency were selected for use intransient knockdown experiments. And siNC was used as a negative control. The siRNAs were transfected using lipofectamine 2000 (Invitrogen, USA) according to the manufacturer’s instructions. The sequences of the siRNAs are listed in Supplementary Table 1.

### Lentivirus packaging and infection

For overexpression, full AC073352.1 cDNA was inserted into the lentivirus expression vector pLent-EF1a-FH-CMV-GFP-P2A-puro, and an empty vector was used for a transfection control group. For knockdown, a lentivirus short hairpin RNA expression vector targeting the same sequence as siAC073352.1-1 was constructed by Obio Technology (Shanghai, China). Next, HEK293T packaging cells were transfected using a retroviral mechanism. Culture supernatants were harvested at 72 h after transfection and infected of BC cells. Stably transfected cells were established using 1 μg/ml puromycin treatment for 2 weeks and were bulk cultured for subsequent assays.

### Quantitative reverse transcription-polymerase chain reaction (qRT-PCR)

Total RNA was isolated from cultured cells using TRIzol (Invitrogen, USA) according to the manufacturer’s instructions. First-strand cDNA was synthesized using the PrimeScript RT Reagent Kit (Takara, Dalian, China). Real-time PCR analyses were performed with SYBR Premix Ex Taq (Takara). The sequences for the gene-specific primers used are listed in Supplementary Table 1. β-Actin was used as an internal control.

### Cell migration and invasion assays

Cell migration and invasion assays were performed using a 24-well plate and 8-μm pore size chamber inserts (Corning, USA), which were precoated with or without 50 μl Matrigel (BD Biosciences, USA). In both assays, cells harvested in 200 μl serum-free media were seeded into the upper chamber and 600 μl medium supplemented with 20% FBS was added to the lower chamber. For migration assays, 5 × 10^4^ cells were seeded into the upper chamber without Matrigel. In addition, for invasion assays, 1 × 10^5^ cells were suspended in the upper chamber with Matrigel. After 36 h of incubation at 37 °C and 5% CO_2_, the cells on the lower surface of the membrane were fixed with 4% paraformaldehyde and stained by Giemsa’s stain. The images were captured with an inversion microscope (Zeiss, Germany) and counting was undertaken using the ImageJ software.

### Wound-healing assay

To investigate their metastatic ability, cells (2 × 10^5^ per well) were seeded in 24-well plates and cultured for 12 h until the confluency of monolayers reached 90–100%. Further, a 200-μl sterile pipette tip was used to scratch across the center of each well before the medium was replaced by new medium supplemented with 2% FBS. After incubating for 0 and 24 h, images of cells in the wells were captured by an inversion microscope (Zeiss, Germany). The wound area was calculated to estimate the cell’s migration efficacy.

### Cell viability assay

Cell proliferation efficacy was determined using the Cell Counting Kit 8 (CCK-8; Bestbio, Shanghai, China). A total of 3000 cells with corresponding treatment were seeded in 96-well plates. Cell viability was measured every 24 h, whereby 10 μl of CCK-8 solution was used for incubation for 2 h at 37 °C and the spectrophotometric absorbance at OD450 was determined by the SpectraMax i3X (Molecular Devices, USA).

### EdU (5-ethynyl-2′-deoxyuridine) incorporation assay

The transfected BC cell lines were incubated in 96-well plates for 24 h, and the EdU Kit (RiboBio, Guangzhou, China) was used following the manufacturer’s instructions. DAPI (4′,6-diamidino-2-phenylindole) was applied to stain cell nuclei (Solarbio, China), and images were obtained to identify positively proliferative cells by use of a fluorescence microscope (Zeiss, Germany).

### RNA fluorescence in situ hybridization

An AC073352.1 probe was obtained from Shanghai Qutdo Biotech. RNA fluorescence in situ hybridization (FISH) for cells on coverslips was performed following the manufacturer’s protocol. Briefly, the cells were washed, fixed, and treated by 0.5% Triton. Next, cells on coverslips were incubated with each specific probe overnight before the slides were stained with DAPI. Finally, fluorescence images were captured by a confocal microscope (Zeiss, Germany). The sequences of the AC073352.1 probe for FISH is listed in Supplementary Table 1.

### Western blot

Cells were harvested and lysed with Western/IP lysis buffer (Beyotime, Haimen, China) containing the protease inhibitor phenylmethanesulfonylfluoride fluoride (Beyotime). The protein concentration was determined using the Enhanced BCA Protein Assay Kit (Beyotime). Proteins were denatured at 100 °C for 10 min, subjected to sodium dodecyl sulphate–polyacrylamide gel electrophoresis (SDS-PAGE), and transferred to 0.22 mm polyvinylidene fluoride membranes (Merck-Millipore, Darmstadt, Germany). The membranes were blocked using 5% non-fat milk and 1% Tween 20 in phosphate-buffered saline (PBS) for 1 h and incubated with primary antibodies at 4 °C overnight. The membranes were subsequently washed and incubated with the appropriate secondary antibodies at room temperature. Finally, the signals were detected by standard analysis of HRPO-induced chemiluminescence. Details of the antibodies used in western blot are listed in Supplementary Table 1.

### RNA pull-down assay

In vitro, full-length sequences of AC073352.1 were transcribed from their corresponding plasmids using Riboprobe Combination Systems (Promega, USA) before being purified using the RNAclean Kit (Qiagen, DP412) and labeled with desthiobiotinylation overnight (Magnetic RNA-Protein Pull-Down Kit, Thermo). Next, 1 mg protein extracted from MDA-MB-231 cells was incubated with biotin-labeled RNA and streptavidin magnetic beads for 6–12 h at 4 °C. After elution of lncRNA-associated proteins, they were separated by SDS-PAGE and visualized by silver staining. Furthermore, the retrieved proteins were subjected to mass spectrometry (MS) analysis (Novogene, Tianjin, China).

### RNA immunoprecipitation (RIP) assay

A RIP assay was performed by using the Imprint RNA Immunoprecipitation Kit (Millipore, USA) according to the manufacturer’s instructions. In brief, 1 × 10^7^ MDA-MB-231 cells were lysed with RIP lysis buffer, and the lysates were incubated with magnetic beads conjugated to 5 μg anti-YBX1 (Abcam), anti-SNRP70 (positive controls) or anti-lgG (negative controls) for 3–6 h at 4 °C. Immunoprecipitated RNA was eluted, purified, and dissolved in RNase-free water, which was further measured through qRT-PCR analysis of YBX1. Specific primers are listed in Supplementary Table 1.

### Exosomes isolation, characterization, and treatment

Exosomes were isolated from BC cell supernatants by ultracentrifugation. BC cells were initially cultured in DMEM medium supplemented with 10% exosome-free FBS. Cell supernatants were collected after 60 h and centrifuged at 300 × *g* for 10 min, 2000 × *g* for 10 min, and 10,000 × *g* for 30 min, all at 4 °C. The obtained supernatant was filtered through 0.22-μm filter (Millipore) followed by ultracentrifugation at 110,000 × *g* for 90 min at 4 °C. The supernatant was discarded, and exosomes pellets were washed with PBS before a second ultracentrifugation was performed at 110,000 × *g* for 90 min at 4 °C.

The purified exosomes were subjected to the following experiments:

Morphological images of exosomes were analyzed using transmission election microscopy (TEM) at 100 keV, and the size distribution of exosomes was analyzed by the nanoparticle tracking analysis (NTA) software.

To monitor exosome trafficking, a PKH67 Green Fluorescent Cell Linker Kit (Sigma Aldrich, St Louis, USA) was used to label exosomes according to the manufacturer’s instruction. The staining was terminated by addition of 1% bovine serum albumin. The PKH67-labeled exosomes were washed in PBS, precipitated by ultracentrifugation, and resuspended in PBS. Next, exosomes were added to HUVECs and incubated for 12 h. The nuclei were stained with DAPI, and images were obtained by using a Zeiss confocal microscope system.

### Endothelial tube-formation assay

HUVECs were treated with exosomes (MDA-MB-231-vector or MDA-MB-231-AC073352.1) for 12 h. Then pretreated HUVECs were seeded onto a 96-well plate coated with reduced growth factor Matrigel (BD Biosciences) and incubated at 37 °C in 5% CO_2_ for 6 h. Tube-like structures formed by the HUVECs were imaged using a light microscope, and total junction numbers were calculated automatically using the angiogenesis analyzer plugin in the ImageJ software.

### Xenograft model

Five-week-old female BALB/c nude mice were purchased from Weitonglihua (Peking, China) and housed in pathogen-free conditions. For experimental lung metastases assays in the xenograft model, MDA-MB-468 cells were stably transfected with shAC073352.1 or shNC lentiviruses, and a total of 1 × 10^6^ cells were suspended in 0.1 ml sterile PBS before being injected into mice via the lateral tail vein (*n* = 7 in each group). After 6 weeks, these mice were sacrificed by cervical dislocation and the resected lung tissues were harvested, fixed, and embedded in paraffin. Subsequently, the number of metastatic foci in the lung was determined using hematoxylin and eosin (HE) staining. All animal experiments were performed in accordance with safe guidelines and regulations, and protocols were approved by the Institutional Animal Care and the Committee of the Second Hospital of Shandong University.

### Statistical analysis

All the measurement data analyses were performed using GraphPad Prism V6.0 (GraphPad prism, Inc., La Jolla, CA, United States) and SPSS V19.0 (SPSS, Chicago, IL, United States). The significance of differences between groups were assessed using Student’s *t* test or *χ*^2^ test as appropriate. The results are presented as mean ± standard error of mean (SEM) of three independent experiments. A *p* value < 0.05 was considered to be statistically significant.

## Results

### AC073352.1 expression is upregulated in BC and associated with poor outcomes

**Fig. 1:**
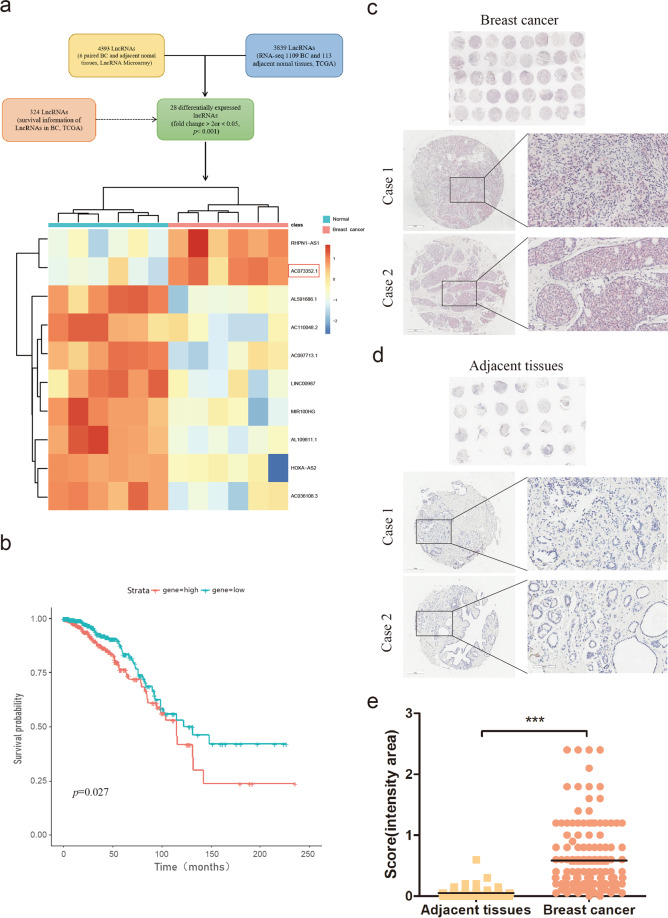
AC073352.1 expression is upregulated and associated with poor outcomes in BC. **a** A flowchart showing the selection of lncRNA AC073352.1 from the LncRNA microarray and TCGA database in BC. A heatmap representation of 10 overlapping lncRNA for gene expression profiles of 6 BC tissues and paired adjacent normal tissues in LncRNA microarray (fold change >2 or <0.5, *p* < 0.001). **b** Kaplan–Meier analyses of the correlation between AC073352.1 RNA levels and the overall survival rate of patients with BC, using TCGA data. Patients were stratified for the analysis using the median. **c**–**e** ISH analysis detecting AC073352.1 expression (red) in BC tissue (*n* = 137) and adjacent normal tissues (*n* = 67). Representative images from **c** two BC cases and **d** adja**c**ent normal tissue are shown (magnification: ×200). **e** Statistical analysis was assess**e**d by *t* test. ***p value < 0.001.

To identify differentially expressed lncRNAs in BC, we performed a lncRNA microarray analysis of six BC tissues and paired adjacent normal breast tissues and analyzed The Cancer Genome Atlas (TCGA) database. Twenty-eight overlapping differentially expressed lncRNAs were found, according to the criteria of a fold change >2 or <0.5 and a *p* < 0.001. Among them, ten candidate lncRNAs were significantly associated with the outcome of BC patients (Supplementary Table 2). Especially, patients with a high AC073352.1 expression had a markedly poorer overall survival compared to those with low AC073352.1 expression; from this, AC073352.1 was selected for further experiments (Fig. 1a, b).

To further validate the clinical significance of AC073352.1 expression, AC073352.1 expression level was detected in 137 BC samples and 67 adjacent normal tissues using ISH. The results showed that AC073352.1 was more highly expressed in BC tissues compared to adjacent normal tissues (Fig. 1c–e). The relationship between AC073352.1 expression and clinical pathological factors was further explored in 137 BC patients. As shown in Supplementary Table 3, greater AC073352.1 expression positively correlated with a high pathological tumor stage (*p* = 0.026) and increased lymph node (LN) metastasis (*p* = 0.006). In summary, this data suggested that AC073352.1 is highly expressed in BC and may serve as a potential prognostic biomarker in the clinic.

To characterize AC073352.1, its sequence and coding potential were comprehensively studied. AC073352.1 (ENSG00000272662.1) is located on chromosome 3q33.13 and it has one 504-bp length transcript (for UCSC Genome browser: http://genome.ucsc.edu/; Supplementary Fig. 1a). The ORF Finder (>300) and Conserved Domain Database from the National Center for Biotechnology Information failed to predict a protein for AC073352.1 (Supplementary Fig. 1b, c). Further investigation using the Coding Potential Assessment Tool and Coding Potential Calculator revealed that AC073352.1 had no coding capability (http://lilab.research.bcm.edu/cpat/index.php and http://cpc2.cbi.pku.edu.cn/; Supplementary Fig. 1d, e).

### AC073352.1 enhances the migration and invasion capabilities of BC cells in vitro

**Fig. 2:**
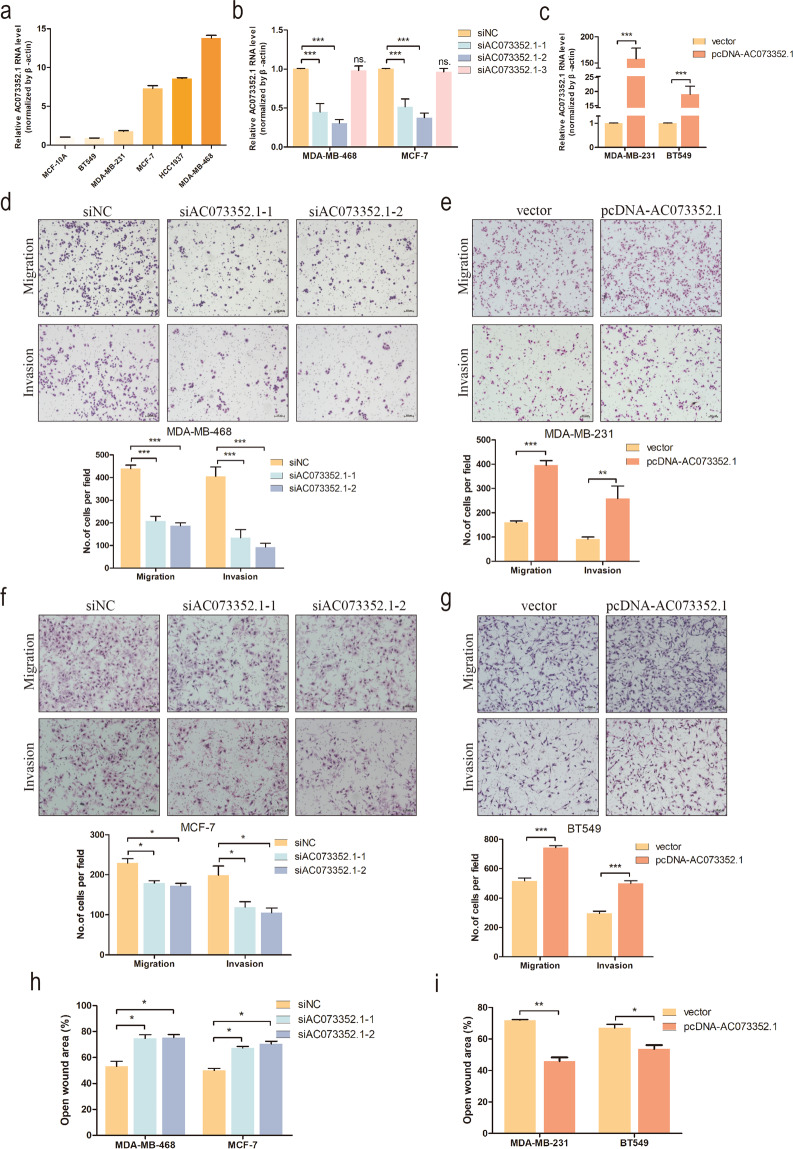
AC073352.1 regulates Breast Cancer cells metastasis in vitro. **a** Expression of AC0733521.1 in the normal breast epithelium cell line (MCF-10A) and BC cell lines was detected by qRT-PCR and normalized to β-actin. **b**, **c** qRT-PCR analysis of AC073352.1 expression in control, siAC073352.-1-, siAC073352.-2-, siAC073352.-3-, and pcDNA-AC073352.1-treated BC cells. **d**, **f** Transwell assays were used to investigate the changes in the migratory and invasive capabilities of MDA-MB-468 and MCF-7 cells after transfection with siAC073352.1s. **e**, **g** Transwell assays were used to investigate the changes in the migratory and invasive capabilities of MDA-MB-231 and BT549 cells after transfection with pcDNA-AC073352.1. **h**, **i** Wound healing assay assessing BC cells’ metastasis. The data represent the mean ± SEM from three independent experiments. Values are expressed as mean ± SEM, *n* = 3. *p < 0.05, **p < 0.01, ***p < 0.001, and ns not significant.

In order to explore the relevance of AC073352.1 expression to BC cells, six cell lines, including one normal mammary epithelial cell line MCF-10A and five BC cell lines (BT549, MDA-MB-231, MCF-7, HCC1937, and MDA-MB-468) were investigated to determine AC073352.1 expression level. As shown in Fig. 2a, AC073352.1 is a widely expressed in different BC cell lines.

Furthermore, to investigate the oncogenic properties of AC073352.1 on BC cells, loss- and gain-of-function experiments were conducted in vitro. MDA-MB-468 and MCF-7 cells with higher intrinsic AC073352.1 level were transfected with siRNAs; siAC073352.1-1 and siAC073352.1-2 were more effective at reducing AC073352.1 level. MDA-MB-231 and BT549 cells, which had lower intrinsic AC073352.1 level, were stably transfected with the overexpression lentiviral vector (pcDNA-AC073352.1) (Fig. 2b, c). The results of transwell assays showed that AC073352.1 knockdown significantly inhibited the migration and invasion capabilities of MDA-MB-468 and MCF-7 cells (Fig. 2d, f); in contrast, the overexpression of AC073352.1 substantially enhanced the migration and invasion capabilities of MDA-MB-231 and BT549 cells (Fig. 2e, g). Similarly, the migration capability of MDA-MB-468 and MCF-7 cells in wound healing assays was markedly decreased after the downregulation of AC073352.1 (Fig. 2h and Supplementary Fig. 2a), the opposite results were found in MDA-MB-231 and BT549 cells that overexpressed AC073352.1 (Fig. 2i and Supplementary Fig. 2b). Neither the CCK-8 assay nor the EdU assay showed that the knockdown and overexpression of AC073352.1 had a significant effect on proliferation of MCF-7 or MDA-MB-231 cells, respectively. (Supplementary Fig. 2c–e).

The above results indicated that AC073352.1 expression improves the migration and invasion capabilities of BC cells in vitro.

### Silencing AC073352.1 inhibits BC metastasis in vivo

**Fig. 3:**
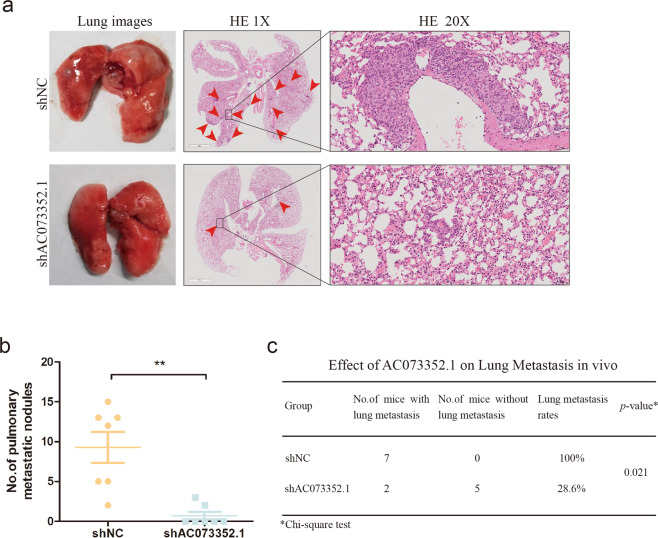
AC073352.1 accelerates breast cancer metastasis in vivo. **a** The representative images of lung metastasis of mice. Hematoxylin and eosin (HE) staining of lungs were removed from mice injected with shNC or shAC073352.1 stable cell line. **b** Metastasis nodules were counted and analyzed using Student *t* test. Values are expressed as mean ± SEM. **c** The ratio of lung metastasis was calculated for the indicated groups. The *p* value was determined using *χ*^2^ test. *p < 0.05 and **p < 0.01.

To further assess the role of AC073352.1 in BC metastasis in vivo, MDA-MB-468 cells were stably transferred with shAC073352.1 or shNC and injected into mouse tail veins to build a metastatic mice model. Metastatic nodules in mice injected with MDA-MB-468 cells were dissected out for HE analysis (Fig. 3a). Compared to the control group, the knockdown of AC073352.1 significantly decreased the number of pulmonary metastatic nodules (Fig. 3b). Metastatic rates were also reduced in the shAC073352.1 group (Fig. 3c). Therefore, this data showed that silencing AC073352.1 significantly impairs the metastasis of BC cells to the lung in vivo.

### AC073352.1 directly binds to YBX1

**Fig. 4:**
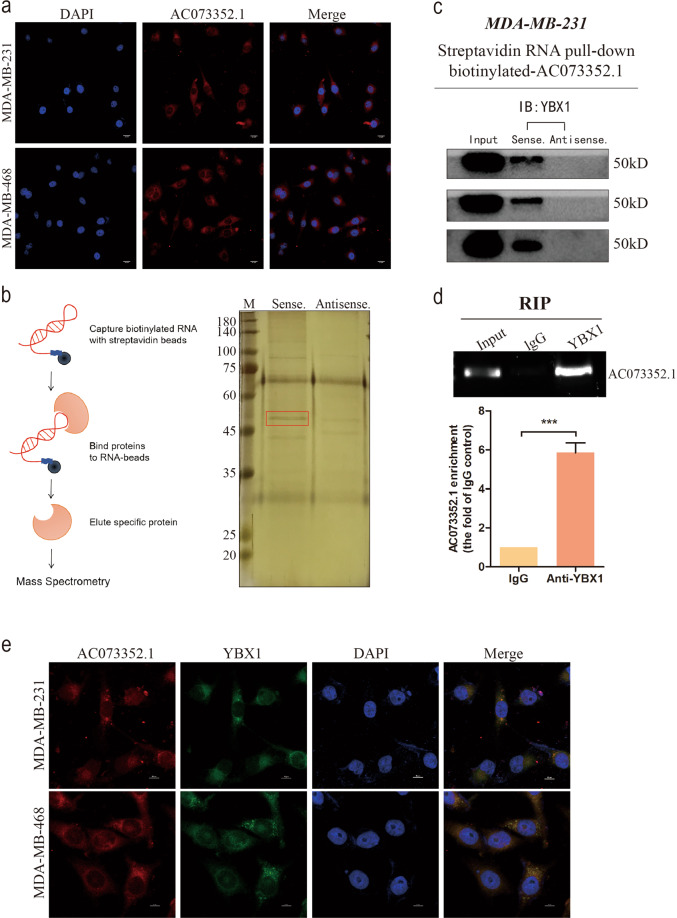
AC073352.1 binds to the transcriptional activator YBX1. **a** AC073352.1 expression in MDA-MB-231 and MDA-MB-468 cells was detected by RNA FISH. Scale bars: 20 μm. **b** AC073352.1 sense and antisense RNAs were used for in vitro transcribed and pull-down assays in MDA-MB-231 cells. After electrophoresis and silver staining, the different bans located at 45–60 kDa. **c** Western blot analysis of the specific association between YBX1 and biotinylated AC073352.1; results obtained from three independent streptavidin RNA pull-down assays. **d** RIP assays were performed using an anti-YBX1 antibody; lgG was used as negative control. Specific primers were used to detect AC073352.1 (*n* = 3). **e** The co-localization of AC073352.1 and YBX1 was assessed by FISH and immunofluorescence. Scale bar: 10 μm. ***p < 0.001.

LncRNAs have been reported to exert their function by interacting with proteins during cancer progression [23]. It was hypothesized that AC073352.1 promotes BC metastasis through protein binding. To confirm this, an RNA FISH assay was performed on BC cells using an AC073352.1 probe (Fig. 4a). Consistent with the ISH (Fig. 1e), the results of this experiment found that the lncRNA exists both in the nucleus and cytoplasm of BC cells. It was mainly distributed in the cytoplasm of the BC cells.

Next, the endogenous binding proteins of AC073352.1 in BC cells were identified using RNA pull-down assays; bound protein was revealed by sliver staining and MS (Fig. 4b). This analysis found 329 proteins that interacted with AC073352.1, including splicing factors and RNA-binding proteins. Among these binding proteins, proteins that correspond to the top ten unique peptides were selected; from this, it was found that YBX1 had the highest levels of interaction with this highest score. This result indicated that AC073352.1 may bind to the transcriptional activator YBX1 (Supplementary Table 4 and Supplementary Fig. 3a). Following this, three independent RNA pull-down complexes were performed, and the results were detected by a western blot analysis using an YBX1 antibody. The results showed that a strong YBX1 signal was observed with the labeled AC073352.1 RNA; this result was not seen when using antisense RNA (Fig. 4c). A RIP assay using the YBX1 antibody also detected the RNA-YBX1 complex and confirmed that YBX1 had significant interaction with AC073352.1 (Fig. 4d). Furthermore, FISH and immunostaining showed that AC073352.1 and YBX1 co-located in BC cells (Fig. 4e), demonstrating that AC073352.1 interacted with the YBX1. These results together indicated that YBX1 specifically interacts with AC073352.1 in BC cells.

### AC073352.1 stabilizes the protein level of YBX1

**Fig. 5:**
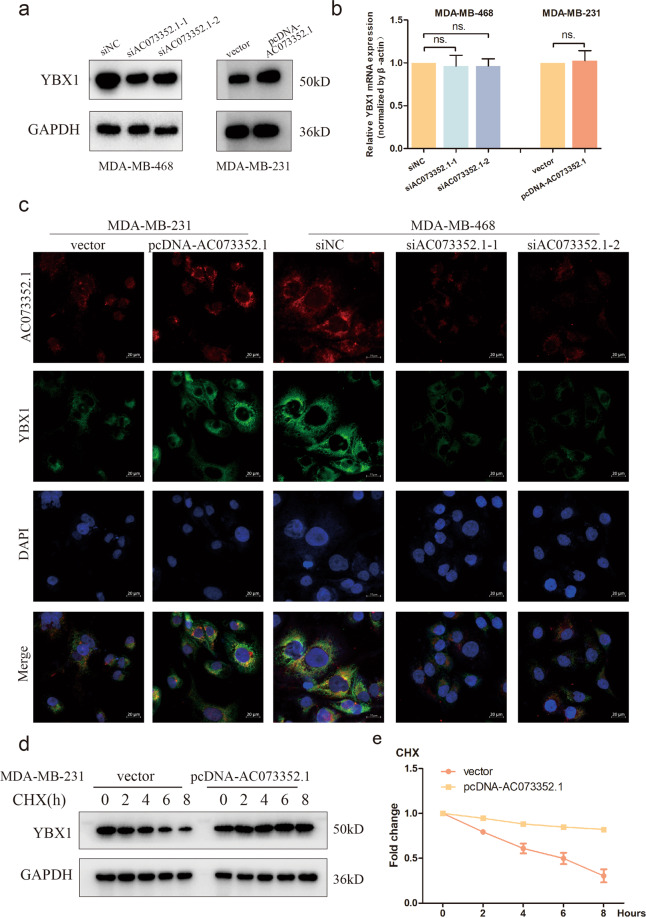
AC073352.1 affects the protein level of YBX1. **a** Western blots analysis showing the protein levels of YBX1 following the knockdown or overexpression of AC073352.1 in BC cells. **b** qRT-PCR analysis showing the mRNA levels of YBX1 mRNA following the knockdown or overexpression of AC073352.1 in BC cells. **c** Expression of YBX1 and AC073352.1 in MDA-MB-468 cells transfected with siNC or AC073352.1 siRNAs and MDA-MB-231 cells transfected with vector or pcDNA-AC073352.1 was analyzed by FISH and immunofluorescence. Scale bar: 20 μm. **d**, **e** The stable overexpression of AC073352.1 in MDA-MB-231 cells treated with cycloheximide (CHX, 200 μg/ml) for the indicated times. **d** Western blot showing YBX1 levels in whole-cell extracts. **e** Densitometry analysis of YBX1 protein levels: the relative fold of the level at 0 h.

Next, qRT-PCR and western blot assays were used to detect whether AC073352.1 regulated the expression of YBX1. Interestingly, it was found that AC077352.1 did not change YBX1 mRNA levels but significantly affected the protein levels. AC073352.1 depletion dramatically reduced the protein expression of YBX1, whereas ectopic overexpression of AC073352.1 elevated YBX1 protein levels in BC cells (Fig. 5a, b). In addition, we performed FISH and immunostaining experiments to assess the expression of YBX1 in BC cells. Consistently, the results showed that the expression level of YBX1 was significantly downregulated after AC073352.1 siRNA treatment in MDA-MB-468 cells, and YBX1 expression was upregulated in MDA-MB-231 cells with AC073352.1 overexpression (Fig. 5c). To further investigate how AC073352.1 affects YBX1 protein stability, YBX1 protein levels were examined in MDA-MB-231 cells overexpressing AC073352.1; these cells had been treated with the protein synthesis inhibitor cycloheximide. As shown in Fig. 5d, e, the protein stability of YBX1 was increased and the half-life was prolonged when AC073352.1 was upregulated in MDA-MB-231 cells. These results indicated that AC073352.1 affects YBX1 expression by increasing its protein stability.

### AC073352.1 promotes migration and invasion of BC cells via YBX1

**Fig. 6:**
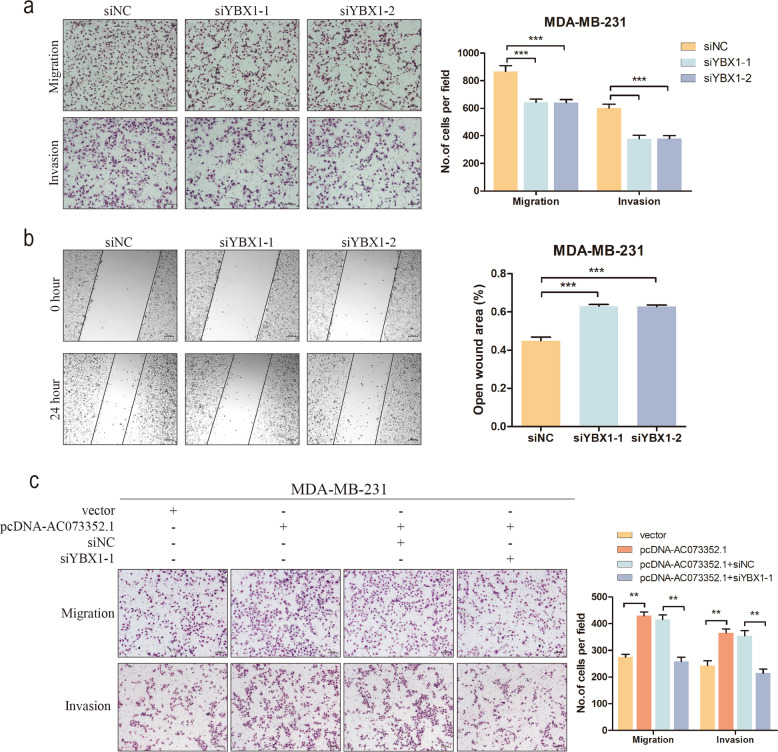
AC073352.1 interacts with the transcriptional activator YBX1 to affect BC metastasis. **a**, **b** Transwell and wound-healing assays on MDA-MB-231 cells transfected with YBX1 siRNA. **c** Rescue assays following transwell assays revealed that the depletion of YBX1 partly reversed the effects of AC073352.1 overexpression in MDA-MB-231 cells. Values are expressed as mean ± SEM, *n* = 3. **p < 0.01, ***p < 0.001.

Existing studies have demonstrated that YBX1 may contribute to the progression of multiple cancers, especially in the context of promoting tumor metastasis [24–26]. Therefore, it was explored whether YBX1 may positively regulate this biological function in BC. We first detected YBX1 expression levels in BC cell lines; the results showed that YBX1 expression were higher in BC cell lines than normal cell line (MCF-10A) (Supplementary Fig. 3d). When YBX1 was effectively silenced (Supplementary Fig. 3e), the migration and invasion capabilities of BC cells was reduced (Fig. 6a, b). Moreover, rescue experiments were used to detect whether the interaction between YBX1 and AC073352.1 contributed to BC metastasis. Unsurprisingly, the knockdown of YBX1 partly reversed the AC073352.1-mediated increase of BC cell migration and invasion (Fig. 6c). Meanwhile, the expression of YBX1 was detected in rescue experiments (Supplementary Fig. 3f). These results further verified that AC073352.1 enhances BC metastasis by interacting with YBX1.

### Exosomal AC073352.1 regulates the angiogenesis of HUVECs via binding YBX1

**Fig. 7:**
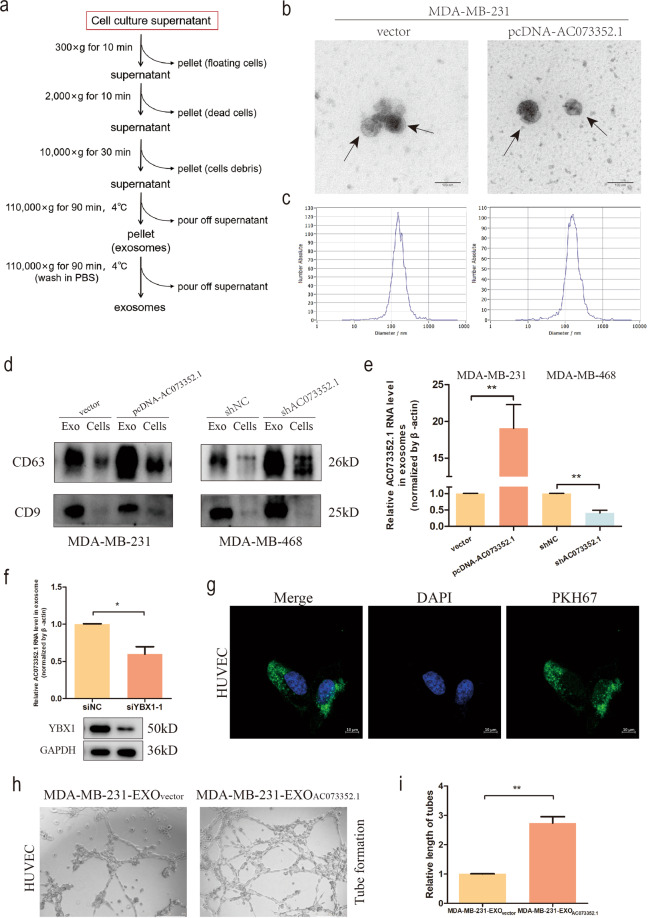
Exosomal AC073352.1 regulates the angiogenesis of HUVECs via binding YBX1. **a** Flow chart for the exosome purification procedure based on differential ultracentrifugation. **b** TEM scanning images of purified exosomes derived from MDA-MB-231 cells overexpressing AC073352.1. Scale bars: 100 nm. **c** Characterization of purified exosomes derived from MDA-MB-231 cells overexpressing AC073352.1. The size, number, and distribution of exosomes was analyzed by NTA. **d** Exosomal protein marker (CD63 and CD9) detection by western blot from purified exosomes and exosome-depleted cell extracts. **e** qRT-PCR analysis of AC073352.1 levels in exosomes from AC073352.1-overexpressing MDA-MB-231 cells and shAC073352.1-treated MDA-MB-468 cells. **f** qRT-PCR analysis of AC073352.1 levels: AC073352.1 levels were decreased in exosomes after MDA-MB-231 cells were transfected with a YBX1 siRNA. **g** HUVECs were incubated with PKH67-lableled exosomes for intercellular trafficking. Scale bars: 10 μm. **h**, **i** Representative images and quantification of tube-formation assays by HUVECs treated with MDA-MB-231_vector_ or MDA-MB-231_AC073352.1_. Scale bars: 100 μm. *p < 0.05 and **p < 0.01.

As exosomes are key mediators of cell–cell communication in the promotion of cancer progression, this experiment aimed to explore the exosome-based mechanism of BC progression. To examine whether AC073352.1 exists in BC cells derived from exosomes, BC-derived exosomes were isolated and characterized. Exosomes purified from the cell culture supernatant of BC cells (Fig. 7a) exhibited a round-shaped morphology and a size ranging from 30 to 150 nm according to TEM and NTA (Fig. 7b, c). A western blot assay further verified the presence of well-defined exosome markers CD63 and CD9 (Fig. 7d). These results indicated that the exosomes were isolated successfully.

Additionally, to explore whether the expression of AC073352.1 in exosomes from stable BC cell lines was altered, qRT-PCR analysis was performed. As expected, when compared to the control group, the expression level of exosomal AC073352.1 was significantly higher in MDA-MB-231 cells overexpressing AC073352.1; these levels were significantly lower in MDA-MB-468 cells treated with shAC073352.1 (Fig. 7e). Previous studies have shown that YBX1 could participate in the packaging of RNAs into exosomes [27]. To clarify whether YBX1 is essential for the loading of AC073352.1 into exosomes, qRT-PCR was performed and it was found that silencing YBX1 decreased the expression levels of exosomal AC073352.1 (Fig. 7f).

Several studies have shown that tumor-derived exosomes can be internalized by recipient cells to achieve their function. Therefore, this experiment focused on “tumor cell–endothelial cell” communication, and it was hypothesized that exosomes derived from BC cells may be internalized by HUVECs, as well as playing a role in the angiogenesis of HUVECs. As expected, HUVECs exhibited an uptake of PKH67-labeled exosomes derived from MDA-MB-231 cells (Fig. 7g), suggesting that exosomes derived from BC cells could be effectively internalized by HUVECs. Furthermore, tube-formation assays revealed that exosomes derived from AC073352.1-overexpressing MDA-MB-231 cells increased HUVEC angiogenesis compared to control group exosomes (Fig. 7h, i). Taken together, these results suggested that lncRNA AC073352.1 is loaded into exosomes via YBX1 and promotes the angiogenic ability of HUVECs.

## Discussion

With the improvement of diagnosis and treatment methods, the overall survival rate of BC has been improved [1]. However, the prognosis for patients with metastases is still poor, with metastasis the major cause of death among BC patients [28]. Hence, there remains a considerable need to explore the underlying molecular mechanisms of BC metastasis and identify novel molecular therapeutic markers. So far, a large number of studies have found that, just like protein-coding genes, lncRNAs act as important players in the development of disease; this has become especially evident in tumor metastasis, including BC. For example, Xiu et al. revealed that the lncRNA LINC02273 could promote BC metastasis via the hnRNPL–AGR2 axis [29]. Zheng et al. demonstrated that a novel triple-negative BC LN metastasis-associated lncRNA, HUMT, can activate FOXK1 transcription to induce BC proliferation and metastasis [30]. In this study, AC073352.1, located at chromosome 3q33.13, was identified in a microarray analysis and TCGA database as one of the several different and novel lncRNAs in BC. The lncRNA AC073352.1 was significantly overexpressed in BC tissues and displayed a positive correlation with lymphatic metastasis and advanced disease in BC. It was important that the higher levels of AC073352.1 associated with a lower overall survival rate in BC patients. Additionally, with GTEx data, it was found that AC073352.1 expression was lower in mammary tissues, which further confirmed that AC073352.1 may play a critical role in BC progression (Supplementary Fig. 1f). In support of this notion, further functional experiments were conducted in vitro and in vivo. The results showed that AC073352.1 promoted BC cell migration and invasion in vitro; in addition to this, silencing AC073352.1 markedly inhibited BC cell lung metastasis in vivo. Taken together, these results provide key evidence that AC073352.1 plays a critical role in BC metastasis and serves as a potential prognostic and therapeutic target for BC metastatic.

Multiple investigations have demonstrated that lncRNAs can perform their biological functions via protein interactions [31, 32]. In this study, RNA pull-down was utilized to identify that transcription factor YBX1 binds to AC073352.1 in BC. RIP and FISH assays further confirmed the binding of AC073352.1 and YBX1. Subsequently, a series of AC073352.1 deletion mapping analysis was detected to further map the specific YBX1-binding region. And we found that the YBX1-binding site may be mainly distributed in the 252–272 nucleotide region of AC073352.1. (Supplementary Fig. 3b, c). More work is needed to prove this in the future. In addition, this study investigated the subcellular fractionation location of AC073352.1, with the results showing that AC073352.1 exists both in the nucleus and cytoplasm of BC cells and it was mainly distributed in BC cell cytoplasm. Growing evidence has indicated that cytoplasmic lncRNAs may regulate protein stability and modification [33, 34]. In consistency with this, it was found that AC073352.1 affected YBX1 protein expression without influencing mRNA levels. In addition, the overexpression of AC073352.1 increased the stability of YBX1 at a protein level. These results suggest that AC073352.1 interacts with YBX1 and stabilizes the protein. Previous studies have shown that lncRNAs regulate the stability of protein via diverse mechanisms, such as ubiquitination, phosphorylation, and other methods at a posttranscriptional level [35, 36]. However, how AC073352.1 affects the protein stability of YBX1 needs to be further explored.

YBX1, a key transcription factor, is located on chromosome 1 (1p34), and as a multi-functional RNA-binding protein, it is highly overexpressed in a variety of tumors; it is established to play a role in several cellular processes, including tumor metastasis [37–41]. To explore whether YBX1 affects cell metastasis in BC, the YBX1 protein was knocked down using siRNAs. The results of this experiment found that silencing YBX1 inhibits BC cell migration and invasion; this may partially rescue AC073352.1-induced BC metastasis. Thus, our results indicated that AC073352.1 mediates cell metastasis by interacting with YBX1 in BC. In recent years, researchers paid special attention that microcalcifications presented in the breast microenvironment and osteoclast-like cells played an important role in BC bone metastases [42, 43]. Previous studies have reported that dysregulated YBX1 is associated with chordoma, osteosarcoma, synovial sarcoma, and other bone diseases [44–47]. Hence, it is a suppose that AC073352.1 may create a favorable condition for bone metastasis by YBX1 in BC. Further studies are needed to identify the detailed mechanisms. It was noted that lncRNA may be directly and indirectly related to gene regulation in BC; in addition to YBX1, other genes could participate in AC073352.1-associated biological function; however, more evidence is needed to confirm this.

Exosomes are a class of small macrovesicles ranging from 30 to 150 nm and are a critical means of exchanging intercellular information that have aroused great research interest [48, 49]. Interestingly, several studies suggest that YBX1 is an important RNA-binding protein that can be secreted into the extracellular matrix, including tumor cell exosomes [50, 51]. Therefore, this investigation tried to explore whether AC073352.1 is incorporated into exosomes by binding YBX1. The results showed that knocking down YBX1 decreased the level of AC073352.1, which indicated that YBX1 may aid in the loading of AC073352.1 into exosomes. Further results showed that exosomes isolated from BC cell supernatant could be internalized by HUVECs and exosomal AC073352.1 promoted angiogenesis. As a point of great interest, this may be another key way that AC073352.1 promotes BC metastasis via YBX1 binding. This data strongly suggests that AC073352.1 may act not only as an intracellular lncRNA in BC metastasis but also as an exosomal lncRNA; this will aid in the identification of novel therapeutic targets for BC.

**Fig. 8:**
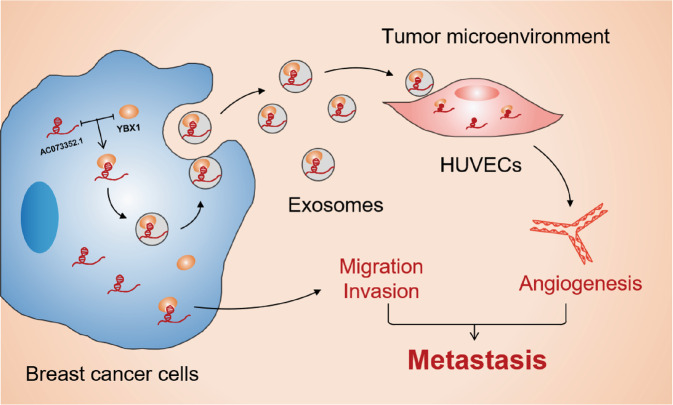
A schematic diagram of the proposed mechanisms. A schematic illustration for the mechanisms by which AC073352.1 interacts with YBX1 to promote BC metastasis and how extracellular AC073352.1 from breast cancer cells was packaged into exosomes and induced HUVEC angiogenesis.

In conclusion, this study identified and characterized a novel lncRNA, AC073352.1, which was significantly overexpressed in BC tissues and associated with poor survival in BC patients. It was also shown that AC073352.1 promotes BC cell metastasis by physically interacting with YBX1 and stabilizing YBX1 at a protein level in BC cells. Moreover, AC073352.1 was transferred into exosomes via YBX1 binding, while exogenous AC073352.1 promotes angiogenesis. These findings illustrate that AC073352.1 promotes BC cell metastasis by functioning within cells and changing the tumor microenvironment (Fig. 8). Collectively, this data offers a mechanistic focus on the oncogenic roles of AC073352.1 and it may serve as a prognostic and therapeutic biomarker for BC.

## Supplementary information

Supplementary Figure Legends

Figure S1

Figure S2

Figure S3

Supplementary Tables

## References

[1] Sung H, Ferlay J, Siegel RL, Laversanne M, Soerjomataram I, Jemal A, et al. Global cancer statistics 2020: GLOBOCAN estimates of incidence and mortality worldwide for 36 cancers in 185 countries. CA Cancer J Clin. 2021;71:209–49. 10.3322/caac.21660 33538338

[2] DeSantis CE, Ma J, Gaudet MM, Newman LA, Miller KD, Goding Sauer A (2019). Breast cancer statistics, 2019. CA Cancer J Clin.

[3] Fan L, Strasser-Weippl K, Li JJ, St Louis J, Finkelstein DM, Yu KD (2014). Breast cancer in China. Lancet Oncol.

[4] Sung H, Ren J, Li J, Pfeiffer RM, Wang Y, Guida JL (2018). Breast cancer risk factors and mammographic density among high-risk women in urban China. NPJ Breast Cancer.

[5] Collaborative Group on Hormonal Factors in Breast Cancer. (2012). Menarche, menopause, and breast cancer risk: individual participant meta-analysis, including 118 964 women with breast cancer from 117 epidemiological studies. Lancet Oncol.

[6] Chaffer CL, Weinberg RA (2011). A perspective on cancer cell metastasis. Science.

[7] Lambert AW, Pattabiraman DR, Weinberg RA (2017). Emerging biological principles of metastasis. Cell.

[8] Ren B, Cui M, Yang G, Wang H, Feng M, You L (2018). Tumor microenvironment participates in metastasis of pancreatic cancer. Mol Cancer.

[9] Evans JR, Feng FY, Chinnaiyan AM (2016). The bright side of dark matter: lncRNAs in cancer. J Clin Investig.

[10] Wang KC, Chang HY (2011). Molecular mechanisms of long noncoding RNAs. Mol Cell.

[11] Quinn JJ, Chang HY (2016). Unique features of long non-coding RNA biogenesis and function. Nat Rev Genet.

[12] Lee S, Kopp F, Chang TC, Sataluri A, Chen B, Sivakumar S (2016). Noncoding RNA NORAD regulates genomic stability by sequestering PUMILIO proteins. Cell.

[13] Wilusz JE, Sunwoo H, Spector DL (2009). Long noncoding RNAs: functional surprises from the RNA world. Genes Dev.

[14] Huang X, Xie X, Liu P, Yang L, Chen B, Song C (2018). Adam12 and lnc015192 act as ceRNAs in breast cancer by regulating miR-34a. Oncogene.

[15] Wang P, Xue Y, Han Y, Lin L, Wu C, Xu S (2014). The STAT3-binding long noncoding RNA lnc-DC controls human dendritic cell differentiation. Science.

[16] Gong P, Qiao F, Wu H, Cui H, Li Y, Zheng Y (2018). LncRNA UCA1 promotes tumor metastasis by inducing miR-203/ZEB2 axis in gastric cancer. Cell Death Dis.

[17] Li P, Zhang X, Wang L, Du L, Yang Y, Liu T (2017). lncRNA HOTAIR contributes to 5FU resistance through suppressing miR-218 and activating NF-kappaB/TS signaling in colorectal cancer. Mol Ther Nucleic Acids.

[18] Zhang L, Niu H, Ma J, Yuan BY, Chen YH, Zhuang Y (2019). The molecular mechanism of LncRNA34a-mediated regulation of bone metastasis in hepatocellular carcinoma. Mol Cancer.

[19] Niu Y, Bao L, Chen Y, Wang C, Luo M, Zhang B (2020). HIF2-induced long noncoding RNA RAB11B-AS1 promotes hypoxia-mediated angiogenesis and breast cancer metastasis. Cancer Res.

[20] Liang Y, Song X, Li Y, Chen B, Zhao W, Wang L (2020). LncRNA BCRT1 promotes breast cancer progression by targeting miR-1303/PTBP3 axis. Mol Cancer.

[21] He L, Zhu W, Chen Q, Yuan Y, Wang Y, Wang J (2019). Ovarian cancer cell-secreted exosomal miR-205 promotes metastasis by inducing angiogenesis. Theranostics.

[22] Chen C, Luo Y, He W, Zhao Y, Kong Y, Liu H (2020). Exosomal long noncoding RNA LNMAT2 promotes lymphatic metastasis in bladder cancer. J Clin Investig.

[23] Wang S, Liang K, Hu Q, Li P, Song J, Yang Y (2017). JAK2-binding long noncoding RNA promotes breast cancer brain metastasis. J Clin Investig.

[24] Li D, Liu X, Zhou J, Hu J, Zhang D, Liu J (2017). Long noncoding RNA HULC modulates the phosphorylation of YB-1 through serving as a scaffold of extracellular signal-regulated kinase and YB-1 to enhance hepatocarcinogenesis. Hepatology.

[25] Lim JP, Shyamasundar S, Gunaratne J, Scully OJ, Matsumoto K, Bay BH (2017). YBX1 gene silencing inhibits migratory and invasive potential via CORO1C in breast cancer in vitro. BMC Cancer.

[26] Lim JP, Nair S, Shyamasundar S, Chua PJ, Muniasamy U, Matsumoto K (2019). Silencing Y-box binding protein-1 inhibits triple-negative breast cancer cell invasiveness via regulation of MMP1 and beta-catenin expression. Cancer Lett.

[27] Shurtleff MJ, Yao J, Qin Y, Nottingham RM, Temoche-Diaz MM, Schekman R (2017). Broad role for YBX1 in defining the small noncoding RNA composition of exosomes. Proc Natl Acad Sci USA.

[28] Steeg PS (2016). Targeting metastasis. Nat Rev Cancer.

[29] Xiu B, Chi Y, Liu L, Chi W, Zhang Q, Chen J (2019). LINC02273 drives breast cancer metastasis by epigenetically increasing AGR2 transcription. Mol Cancer.

[30] Zheng S, Yang L, Zou Y, Liang JY, Liu P, Gao G (2020). Long non-coding RNA HUMT hypomethylation promotes lymphangiogenesis and metastasis via activating FOXK1 transcription in triple-negative breast cancer. J Hematol Oncol.

[31] Wen Z, Lian L, Ding H, Hu Y, Xiao Z, Xiong K (2020). LncRNA ANCR promotes hepatocellular carcinoma metastasis through upregulating HNRNPA1 expression. RNA Biol.

[32] Ferre F, Colantoni A, Helmer-Citterich M (2016). Revealing protein-lncRNA interaction. Brief Bioinform.

[33] Zhuo W, Liu Y, Li S, Guo D, Sun Q, Jin J (2019). Long noncoding RNA GMAN, up-regulated in gastric cancer tissues, is associated with metastasis in patients and promotes translation of Ephrin A1 by competitively binding GMAN-AS. Gastroenterology.

[34] Li YP, Duan FF, Zhao YT, Gu KL, Liao LQ, Su HB (2019). A TRIM71 binding long noncoding RNA Trincr1 represses FGF/ERK signaling in embryonic stem cells. Nat Commun.

[35] Liu J, Liu ZX, Wu QN, Lu YX, Wong CW, Miao L (2020). Long noncoding RNA AGPG regulates PFKFB3-mediated tumor glycolytic reprogramming. Nat Commun.

[36] Yu T, Zhao Y, Hu Z, Li J, Chu D, Zhang J (2017). MetaLnc9 facilitates lung cancer metastasis via a PGK1-Activated AKT/mTOR pathway. Cancer Res.

[37] Su W, Feng S, Chen X, Yang X, Mao R, Guo C (2018). Silencing of long noncoding RNA MIR22HG triggers cell survival/death signaling via oncogenes YBX1, MET, and p21 in lung cancer. Cancer Res.

[38] Goodarzi H, Liu X, Nguyen HC, Zhang S, Fish L, Tavazoie SF (2015). Endogenous tRNA-derived fragments suppress breast cancer progression via YBX1 displacement. Cell.

[39] Peng Z, Wang J, Shan B, Li B, Peng W, Dong Y (2018). The long noncoding RNA LINC00312 induces lung adenocarcinoma migration and vasculogenic mimicry through directly binding YBX1. Mol Cancer.

[40] Shinkai K, Nakano K, Cui L, Mizuuchi Y, Onishi H, Oda Y (2016). Nuclear expression of Y-box binding protein-1 is associated with poor prognosis in patients with pancreatic cancer and its knockdown inhibits tumor growth and metastasis in mice tumor models. Int J Cancer.

[41] Kwon E, Todorova K, Wang J, Horos R, Lee KK, Neel VA (2018). The RNA-binding protein YBX1 regulates epidermal progenitors at a posttranscriptional level. Nat Commun.

[42] Clemenceau A, Michou L, Diorio C, Durocher F. Breast cancer and microcalcifications: an osteoimmunological disorder? Int J Mol Sci. 2020;21:8613. 10.3390/ijms21228613 PMC7696282 33203195

[43] Cox RF, Hernandez-Santana A, Ramdass S, McMahon G, Harmey JH, Morgan MP (2012). Microcalcifications in breast cancer: novel insights into the molecular mechanism and functional consequence of mammary mineralisation. Br J Cancer.

[44] Liang C, Ma Y, Yong L, Yang C, Wang P, Liu X (2019). Y-box binding protein-1 promotes tumorigenesis and progression via the epidermal growth factor receptor/AKT pathway in spinal chordoma. Cancer Sci.

[45] Chatterjee M, Rancso C, Stühmer T, Eckstein N, Andrulis M, Gerecke C (2008). The Y-box binding protein YB-1 is associated with progressive disease and mediates survival and drug resistance in multiple myeloma. Blood.

[46] Oda Y, Ohishi Y, Saito T, Hinoshita E, Uchiumi T, Kinukawa N (2003). Nuclear expression of Y-box-binding protein-1 correlates with P-glycoprotein and topoisomerase II alpha expression, and with poor prognosis in synovial sarcoma. J Pathol.

[47] Oda Y, Sakamoto A, Shinohara N, Ohga T, Uchiumi T, Kohno K (1998). Nuclear expression of YB-1 protein correlates with P-glycoprotein expression in human osteosarcoma. Clin Cancer Res.

[48] Li R, Wang Y, Zhang X, Feng M, Ma J, Li J, et al. Exosome-mediated secretion of LOXL4 promotes hepatocellular carcinoma cell invasion and metastasis. Mol Cancer. 2019;18:18. 10.1186/s12943-019-0948-8 PMC6354392 30704479

[49] Han K, Wang FW, Cao CH, Ling H, Chen JW, Chen RX (2020). CircLONP2 enhances colorectal carcinoma invasion and metastasis through modulating the maturation and exosomal dissemination of microRNA-17. Mol Cancer.

[50] Suresh PS, Tsutsumi R, Venkatesh T (2018). YBX1 at the crossroads of non-coding transcriptome, exosomal, and cytoplasmic granular signaling. Eur J Cell Biol.

[51] Shurtleff MJ, Temoche-Diaz MM, Karfilis KV, Ri S, Schekman R. Y-box protein 1 is required to sort microRNAs into exosomes in cells and in a cell-free reaction. Elife. 2016;5:e19276. 10.7554/eLife.19276 PMC5047747 27559612

