# Supplementary material for: A novel long non-coding RNA AC073352.1 promotes metastasis and angiogenesis via interacting with YBX1 in breast cancer
Source: Cell Death Dis. 2021 Jul 3;12(7):670. doi: 10.1038/s41419-021-03943-x (PMC8254808; doi:10.1038/s41419-021-03943-x)
Supplement: Supplementary file 1 — Supplementary Figure Legends [file 41419_2021_3943_MOESM1_ESM.docx]

**Supplementary Figure Legends**

**Supplementary Fig.1** **The characterization of AC073352.1.** **a** AC073352.1 is located in chromosome 3q13.33 and has only one 504bp transcript (UCSC Genome browser (GRCh38/hg38)). **b** Location and length of ORF in AC073352.1, predicted by ORF Finder. **c** CDD was used to predict the coding potential of AC073352.1. **d**, **e** CPAT and CPC2 predicted a low probability for protein-coding potential for AC073352.1. **f**. Expression of AC073352.1 across diverse normal human tissues from GTEx (https://www.gtexportal.org/home/index.html).

**Supplementary Fig.2** **The effects of AC073352.1 on migration and proliferation of BC cells in vitro.** **a,** **b** Representative graphs of wound healing assay was assessed for BC cells' metastasis. **c** CCK8 assay for MCF-7 cells infected with siRNAs of AC073352.1 or siNC. **d** CCK8 assay for MDA-MB-231 cells infected with the over-expressing AC073352.1 lentivirus or the control. **e** An EdU assay was performed to show the effect of AC073352.1 knockdown or over-expression on cell proliferation. Values are expressed as mean± SEM, n= 3. * p< 0.05, ** p< 0.01. *** p< 0.001. ns. not significant.

**Supplementary Fig.3** **AC073352.1 interacts with YBX1.** **a** The representative MS spectra of the YBX1 peptide and the peptide sequence identified in the sense of AC073352.1 as compared to the antisense. **b** Predicted second structure of AC073352.1 (Annolnc, http://annolnc.cbi.pku.edu.cn/; RNAfold, http://rna.tbi.univie.ac.at/). The red color indicated a strong confidence for the prediction of each base. **c** Deletion mapping of the YBX1-binding domain in AC073352.1. The upper panel: the in vitro, transcribed, full-length, biotinylated AC073352.1 (#1) and truncated, biotinylated, AC073352.1 RNA motifs (#2:1- 272nt; #3:252- 504nt; #4:177- 428nt) showing the correct sizes. The lower panel: western blot of YBX1 in protein samples pull-down by the different AC073352.1 constructs. **d** The relative expression of YBX1 in the normal breast epithelium cell line (MCF-10A) and BC cell lines was detected by western blot. **e** YBX1 protein levels were knocked down in MDA-MB-231 cells after they were transfected with YBX1 siRNAs. **f** Western blot analysis of rescue assays revealed that depletion of YBX1 partly reversed the effects of AC073352.1 over-expression in MDA-MB-231 cells.
