# Supplementary figures and images for: A novel long non-coding RNA AC073352.1 promotes metastasis and angiogenesis via interacting with YBX1 in breast cancer
Source: Cell Death Dis. 2021 Jul 3;12(7):670. doi: 10.1038/s41419-021-03943-x (PMC8254808; doi:10.1038/s41419-021-03943-x)

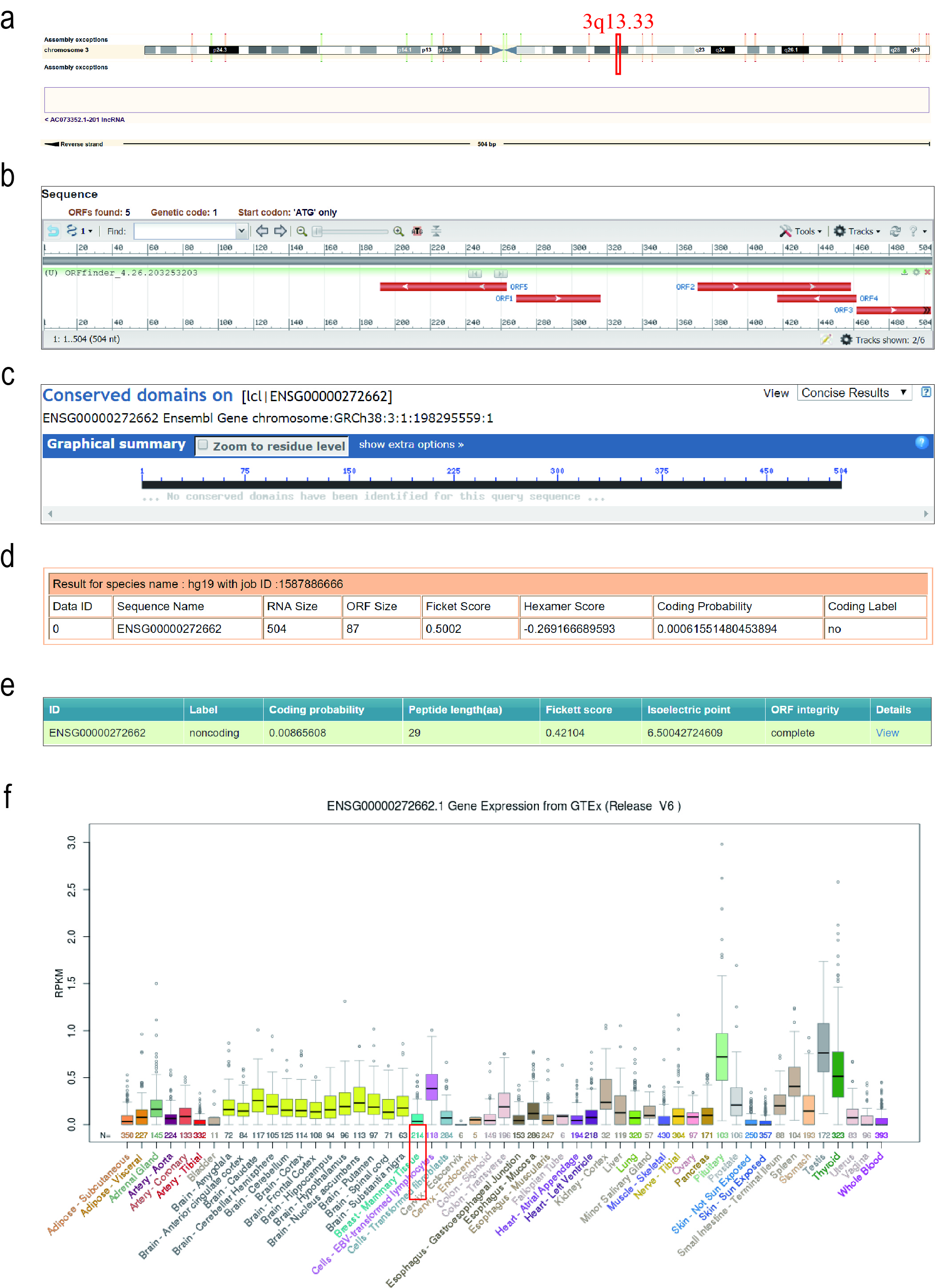

Supplement: Supplementary file 2 — Figure S1 [file 41419_2021_3943_MOESM2_ESM.jpg]

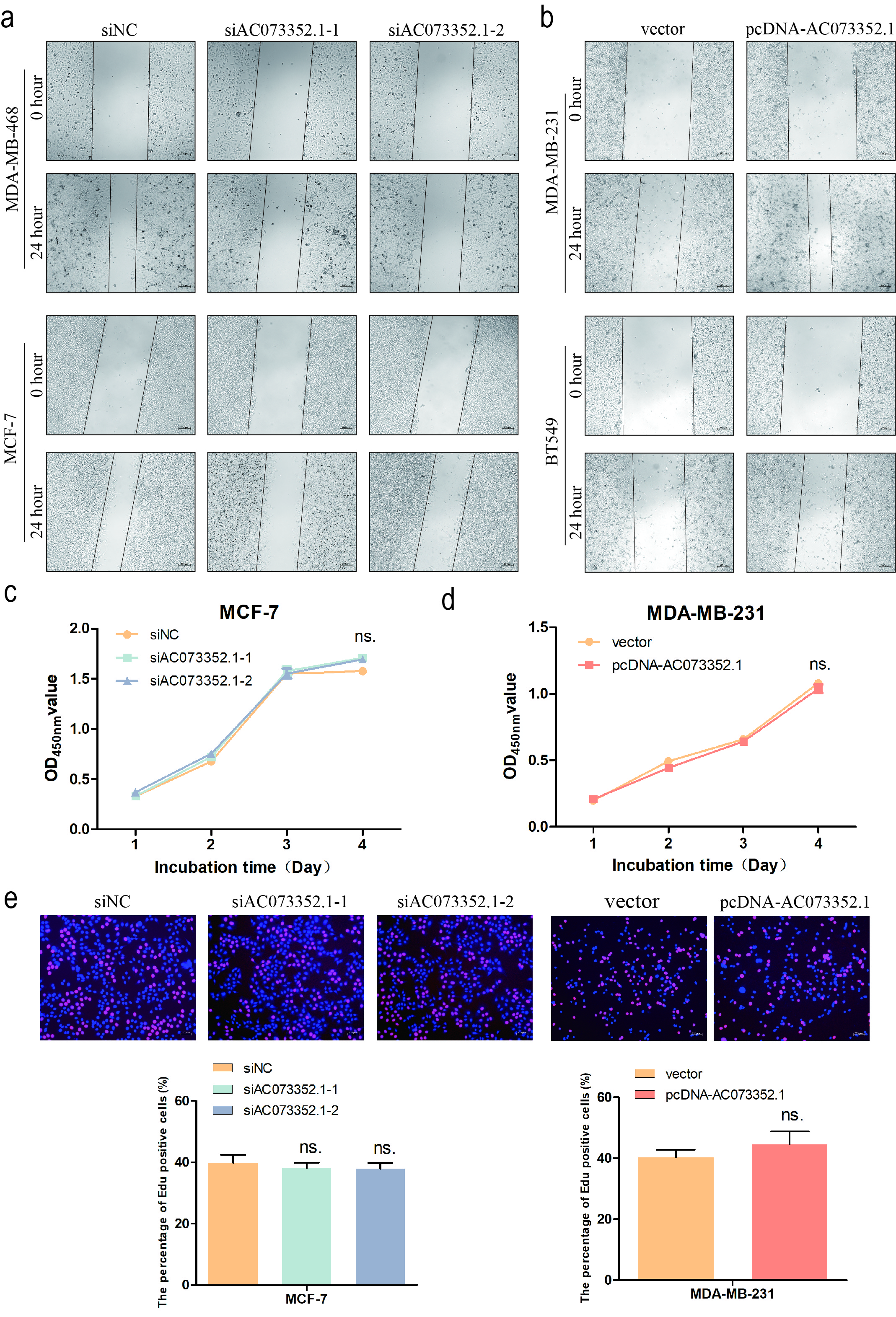

Supplement: Supplementary file 3 — Figure S2 [file 41419_2021_3943_MOESM3_ESM.jpg]

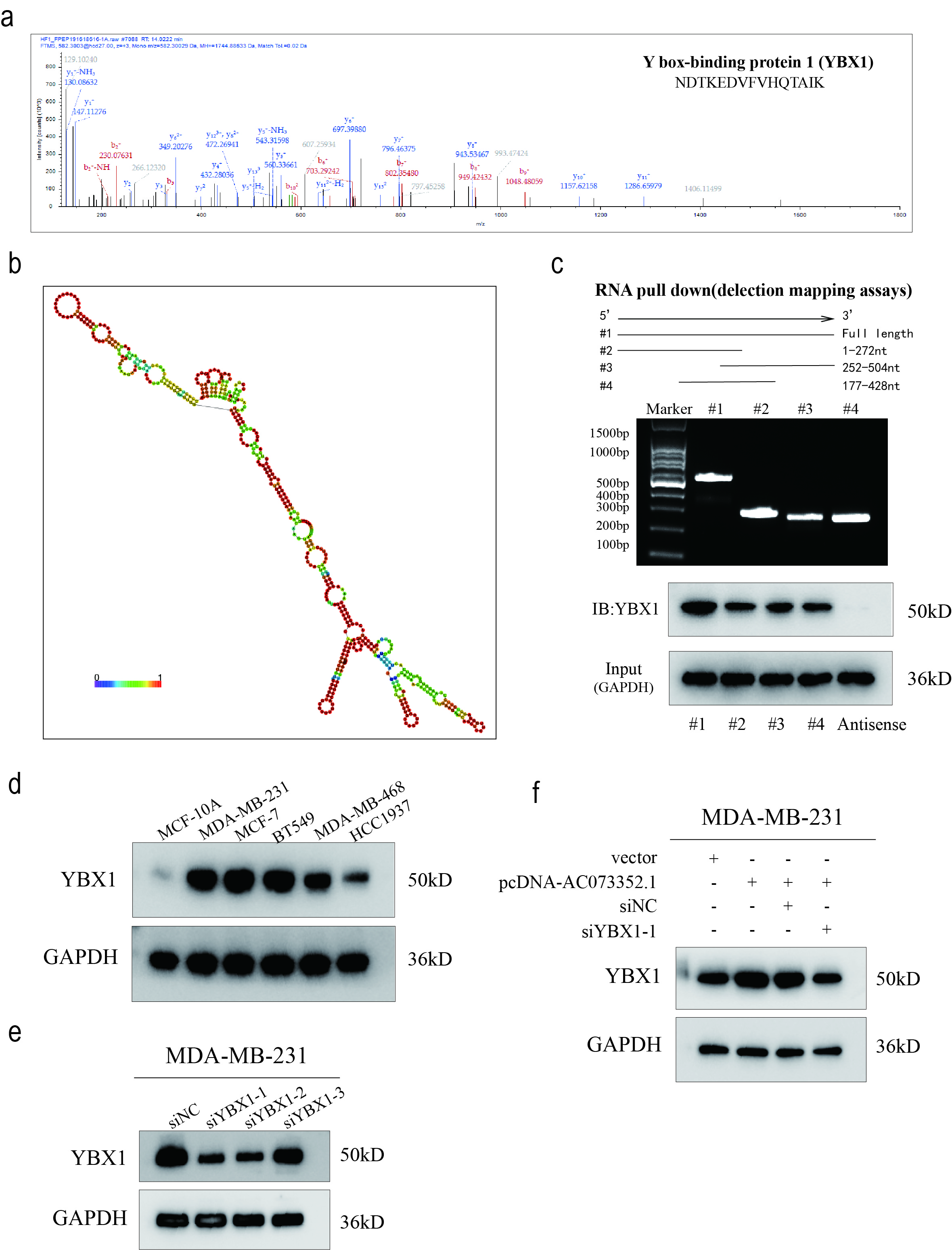

Supplement: Supplementary file 4 — Figure S3 [file 41419_2021_3943_MOESM4_ESM.jpg]
