# Supplementary material for: A novel long non-coding RNA AC073352.1 promotes metastasis and angiogenesis via interacting with YBX1 in breast cancer
Source: Cell Death Dis. 2021 Jul 3;12(7):670. doi: 10.1038/s41419-021-03943-x (PMC8254808; doi:10.1038/s41419-021-03943-x)
Supplement: Supplementary file 5 — Supplementary Tables [file 41419_2021_3943_MOESM5_ESM.docx]

**Supplementary Table 1**. **List of primer, probes, siRNA sequences, and antibody informations.**

| **Primer sequences of target genes** | | |
| --- | --- | --- |
| **Gene** | **Forward primer** | **Reverse primer** |
| β-actin | CATGTACGTTGCTATCCAGGC | CTCCTTAATGTCACGCACGAT |
| AC073352.1 | TGGCAGGTCTTCCTAAGGTG | AGCAGAGGTTGTAGTGACGG |
| YBX1 | GGACAAGAAGGTCATCGCAAC | TCTCCATCTCCTACACTGCGA |
| AC073352.1(1-272) | TTGCAGTGAGCCGAGATCAC | CCATCCCCAATGCCTCGTT |
| AC073352.1(252-504) | AAAACGAGGCATTGGGGAT | TTTTAAATGCATTCATTTTAATG |
| AC073352.1(1-272) | CCTACTCTGCCAACATTAGAG | ACAAACACTTGATAGAAATTCCAG |
| **Probes sequences** | | |
| **Gene** | **Sequence** | |
| AC073352.1-ISH | TGTCACTAATTGAGCTGGTCA | |
| AC073352.1-FISH | CCAGCAGAACTGACAAGGTACAAACGGA | |
| **Sequences for siRNAs** | | |
| **Gene** | **Sequence** | |
| siNC | UUCUCCGAACGUGUCACGUTTACGUGACACGUUCGGAGAATT | |
| siAC073352.1-1 | GGUCUUCCUAAGGUGGAUUTTAAUCCACCUUAGGAAGACCTT | |
| siAC073352.1-2 | GCUAACAUAUGCUUCGAAGTTCUUCGAAGCAUAUGUUAGCTT | |
| siAC073352.1-3 | GCCAGUUACACUAAGAUUUTT AAAUCUUAGUGUAACUGGCTT | |
| siYBX1-1 | GGAGUUUGAUGUUGUUGAATTUUCAACAACAUCAAACUCCTT | |
| siYBX1-2 | GGUUCCCACCUUACUACAUTTAUGUAGUAAGGUGGGAACCTT | |
| siYBX1-3 | GCAGGAGAACAAGGUAGACTTGUCUACCUUGUUCUCCUGCTT | |
| **Antibodies for Immunoblotting, RIP** | | |
| **Protein Name** | **Company** | **Catalog Number** |
| β-actin | Cell Signaling Technology | 3700S |
| GAPDH | Cell Signaling Technology | 5174S |
| YBX1 | Abcam | ab76149 |
| CD63 | Abcam | ab134045 |
| CD9 | Cell Signaling Technology | 13174S |
| Anti-rabbit IgG, HRP-linked Antibody | Cell Signaling Technology | 7074P2 |
| Anti-rabbit IgG, HRP-linked Antibody | Cell Signaling Technology | 7076S |

**Supplementary Table 2. Identificantion of ten lncRNAs candidates from 6 pairs Breast Cancer and adjacent normal tissues**

| GeneSymbol | Location | Gene_ID | pvalues | foldchange | Regulation |
| --- | --- | --- | --- | --- | --- |
| RHPN1-AS1 | Chromosome 8: 143,366,631-143,368,548 | ENSG00000254389 | 7.31E-05 | 3.232247524 | Up |
| AC073352.1 | Chromosome 3: 119,497,678-119,498,181 | ENSG00000272662 | 0.000154074 | 3.114411608 | Up |
| AL591686.1 | Chromosome 1: 242,203,555-242,210,827 | ENSG00000224525 | 2.84E-05 | 0.479370125 | Down |
| AC110048.2 | Chromosome 15: 66,860,303-66,867,023 | ENSG00000277152 | 0.000835926 | 0.407721268 | Down |
| AC097713.1 | Chromosome 2: 234,438,328-234,462,127 | ENSG00000228162 | 0.00099175 | 0.380706388 | Down |
| LINC00987 | Chromosome 12: 9,240,003-9,257,960 | ENSG00000237248 | 0.000565266 | 0.379586535 | Down |
| MIR100HG | Chromosome 11: 122,028,325-122,556,721 | ENSG00000255248 | 7.96E-05 | 0.360139917 | Down |
| AL109811.1 | Chromosome 1: 11,068,471-11,073,097 | ENSG00000226849 | 0.00012143 | 0.13963689 | Down |
| HOXA-AS2 | Chromosome 7: 27,107,777-27,134,302 | ENSG00000253552 | 0.000736114 | 0.113056884 | Down |
| AC036108.3 | Chromosome 15: 99,139,317-99,145,370 | ENSG00000261616 | 0.00010622 | 0.08517795 | Down |

**Supplementary Table 3. Relationships between AC073352.1 expression and clinicopathological** **features of patients with BC**

| **variables** | **LncRNA AC073352.1 expression** | | **total** | **χ2** | **p value** |
| --- | --- | --- | --- | --- | --- |
|  | **Low（n=81）** | **High**  **（n=56）** |  |  |  |
| **Age（years）** |  |  |  | 0.232 | 0.63 |
| <55 | 40 | 30 | 70 |  |  |
| ≥55 | 41 | 26 | 67 |  |  |
| **T stage** |  |  |  | 0.657 | 0.418 |
| T1 | 36 | 21 | 57 |  |  |
| T2/T3 | 45 | 35 | 80 |  |  |
| **N stage** |  |  |  | 7.457 | **0.006** |
| N0 | 51 | 22 | 73 |  |  |
| N1/N2/N3 | 30 | 34 | 64 |  |  |
| **TNM stage** |  |  |  | 4.977 | **0.026** |
| Ι | 25 | 8 | 33 |  |  |
| II/III | 56 | 48 | 104 |  |  |
| **HER2** |  |  |  | 0.002 | 0.965 |
| Negative | 59 | 43 | 102 |  |  |
| Positive | 14 | 10 | 24 |  |  |
| **PR** |  |  |  | 0.405 | 0.525 |
| Negative | 37 | 28 | 65 |  |  |
| Positive | 43 | 26 | 69 |  |  |
| **ER** |  |  |  | 0.92 | 0.337 |
| Negative | 23 | 20 | 43 |  |  |
| Positive | 56 | 34 | 90 |  |  |
| **Pathologic type** |  |  |  | 0.062 | 0.803 |
| Invasive ductal carcinoma | 73 | 52 | 125 |  |  |
| Other | 8 | 4 | 12 |  |  |
| **Grade** |  |  |  | 0.149 | 0.7 |
| II | 69 | 49 | 118 |  |  |
| III | 12 | 7 | 19 |  |  |
| **Lumina type** |  |  |  | 1.221 | 0.748 |
| A | 46 | 28 | 74 |  |  |
| B | 9 | 7 | 16 |  |  |
| HER2 overexpression | 4 | 3 | 7 |  |  |
| Triple negative | 13 | 13 | 26 |  |  |

*Chi-square test

**Supplementary Table 4. A list of top ten potential AC073352.1-interacting protein candidates in MDA-MB-231 cells based on RNA pull-down assays and MS**

| **Accessions** | **Protein name** | **Gene** | **AC073352.1-Sense** | | | | | |
| --- | --- | --- | --- | --- | --- | --- | --- | --- |
|  |  |  | **Coverage [%]** | **PSMs** | **Peptides** | **Unique Peptides** | **Score** | **Abundances (Normalized)** |
| P67809 | Nuclease-sensitive element-binding protein 1 | YBX1 | 35 | 16 | 9 | 9 | 56.22 | 51756435.78125 |
| P11940 | Polyadenylate-binding protein 1 | PABPC1 | 29 | 22 | 18 | 12 | 66.58 | 151714700.5 |
| Q53G85 | Elongation factor 1-alpha | EF1A | 28 | 20 | 9 | 9 | 65.58 | 132550755.125 |
| P26196 | Probable ATP-dependent RNA helicase DDX6 | DDX6 | 27 | 11 | 9 | 9 | 31.73 | 8029534.96875 |
| F8W930 | Insulin-like growth factor 2  mRNA-binding protein 2 | IGF2BP2 | 21 | 11 | 9 | 9 | 33.06 | 10536542.53125 |
| O60506 | Heterogeneous nuclear ribonucleoprotein Q | SYNCRIP | 20 | 15 | 11 | 8 | 47.18 | 83048574.875 |
| V9HW22 | Epididymis luminal protein 33 | HEL-S-72p | 17 | 11 | 9 | 7 | 29.44 | 16735735.2734375 |
| A7BI36 | p180/ribosome receptor | RRBP1 | 11 | 14 | 13 | 13 | 40.47 | 23879520.5625 |
| E5KNY5 | Leucine-rich PPR-motif containing | LRPPRC | 9 | 11 | 11 | 11 | 27.62 | 8933773.5 |
| Q9NZB2 | Constitutive coactivator of  PPAR-gamma-like protein 1 | FAM120A | 9 | 8 | 8 | 8 | 23.48 | 12066244.375 |
